# A population-based study of prevalence and risk factors of chronic kidney disease in León, Nicaragua

**DOI:** 10.1186/s40697-015-0041-1

**Published:** 2015-02-24

**Authors:** Jill F Lebov, Eliette Valladares, Rodolfo Peña, Edgar M Peña, Scott L Sanoff, Efren Castellón Cisneros, Romulo E Colindres, Douglas R Morgan, Susan L Hogan

**Affiliations:** UNC Kidney Center, 7024 Burnett-Womack CB # 7155, Chapel Hill, NC 27599-7155 USA; Centro de Investigaciones e Intervenciones en Salud, León, Nicaragua; Centro de Salud Torre Ramona, Unidad Docente de Medicina Familiar y Comunitaria, Sector II, Zaragoza, España; Duke University, Durham, NC; Department of Physiological Sciences, UNAN-León, Nicaragua; Department of Medicine, Vanderbilt University, Nashville, TN USA; Department of Medicine, UNC Chapel Hill, NC USA

**Keywords:** Chronic kidney disease, Mesoamerican nephropathy, Nicaragua, Central America

## Abstract

**Background:**

Recent studies have shown an excess of chronic kidney disease (CKD) among younger adult males in the Pacific coastal region of Nicaragua and suggest a non-conventional CKD etiology in this region. These studies have been conducted in small, non-representative populations.

**Objectives:**

We conducted a large population-based cross-sectional study to estimate CKD prevalence in León, Nicaragua, and to evaluate the association between previously investigated risk factors and CKD.

**Methods:**

Estimated glomerular filtration rate, derived using the MDRD equation, was assessed to determine CKD status of 2275 León residents. Multivariable logistic regression was used to estimate adjusted prevalence odds ratios. León CKD prevalence was also standardized to the demographic distributions of the León Health and Demographic Surveillance System and the León 2005 Census.

**Results:**

CKD prevalence was 9.1%; twice as high for males (13.8%) than females (5.8%). In addition to gender, older age, rural zone, lower education level, and self-reported high blood pressure, more years of agricultural work, lija (unregulated alcohol) consumption, and higher levels of daily water consumption were significantly associated with CKD. Notably, self-reported diabetes was associated with CKD in adjusted models for females but not males.

**Conclusions:**

Our findings are comparable to those found in regional studies and further support the hypothesis of a Mesoamerican Nephropathy.

**Electronic supplementary material:**

The online version of this article (doi:10.1186/s40697-015-0041-1) contains supplementary material, which is available to authorized users.

## Abrégé

**Contexte:**

Selon de récentes études, il existerait une prévalence d’insuffisance rénale chronique (IRC) excessive chez les jeunes adultes de sexe masculin de la côte du Pacifique du Nicaragua. Ces études, qui ont été conduites sur des échantillons non représentatifs de la population, suggèrent une étiologie non classique de l’IRC dans cette région.

**Objectifs de l’étude:**

Nous avons effectué une étude transversale portant sur un vaste échantillon de population, afin de pouvoir estimer la prévalence d’IRC dans la ville nicaraguéenne de León, d’une part, et évaluer la présence de liens entre l’IRC et certains facteurs de risque ayant été étudiés, d’autre part.

**Méthode:**

Pour déterminer le statut d’IRC de 2275 résidents de la ville de León, nous avons utilisé le débit de filtration glomérulaire estimé, selon l’équation du MDRD. Une régression logistique multivariée a été utilisée pour estimer les ratios de probabilité corrigés de prévalence. La prévalence d’IRC de León a également été normalisée sur la base de la distribution démographique du Health and Demographic Surveillance System de León et avec son recensement de 2005.

**Résultats:**

La prévalence d’IRC était de 9,1%; elle était deux fois plus élevée chez les hommes (13,8%) que chez les femmes (5,8%). D’autres facteurs ont été liés de façon significative à l’IRC: la vieillesse, la vie rurale, un niveau d’éducation faible, une hypertension autodéclarée, plusieurs années de travail en agriculture, et la consommation de lija (alcool non contrôlé) et de grandes quantités d’eau. Le diabète autodéclaré était également lié à l’IRC dans les modèles ajustés chez les femmes, mais non chez les homm es.

**Conclusions:**

Nos résultats sont comparables à ceux des études locales et supportent l’hypothèse de l’existence d’une néphropathie méso-américaine.

## What was known before

Epidemiological studies suggest an elevated prevalence of chronic kidney disease in Pacific Coastal areas of El Salvador and Nicaragua, particularly among male agricultural populations. Prior research in this area has been limited to small sample sizes or mainly rural populations.

## What this adds

The results of this study, conducted among a large population-based sample of residents of the Pacific Coastal municipality of León, Nicaragua, confirm prior findings of high prevalence of CKD in this region and point to the likely contribution of non-traditional risk factors to this burgeoning regional CKD epidemic. Elevated prevalence appears to be concentrated in rural zones and among agricultural workers.

## Background

Several epidemiologic studies have described an excess of chronic kidney disease (CKD) among younger adult males in the Pacific coastal regions of Nicaragua and El Salvador [1-6]. Investigations into potential risk factors for CKD in this region have observed associations between CKD and agricultural work as well as consumption of unregulated alcohol (lija) and water [1-4,6,7]. However, results across studies have been somewhat inconsistent and findings are frequently based on small or non-population-based samples, resulting in analyses with limited statistical power and/or limited generalizability. Additionally, most research has been conducted in rural agricultural communities, while the burden of disease remains largely unknown in more geographically diverse areas.

Given the limited financial and human resources available in the health sector, a valid estimate of CKD prevalence is needed in Nicaragua to inform resource allocation and prevention programs. Moreover, the scarcity and prohibitively high cost of dialysis and kidney transplantation necessitate the identification of upstream risk factors. Using a population-based sample, our primary goal was to estimate the prevalence of CKD in León, Nicaragua. Additionally, we aimed to evaluate the association between previously investigated risk factors and CKD in this region.

## Methods

### Study population

**Figure 1:**
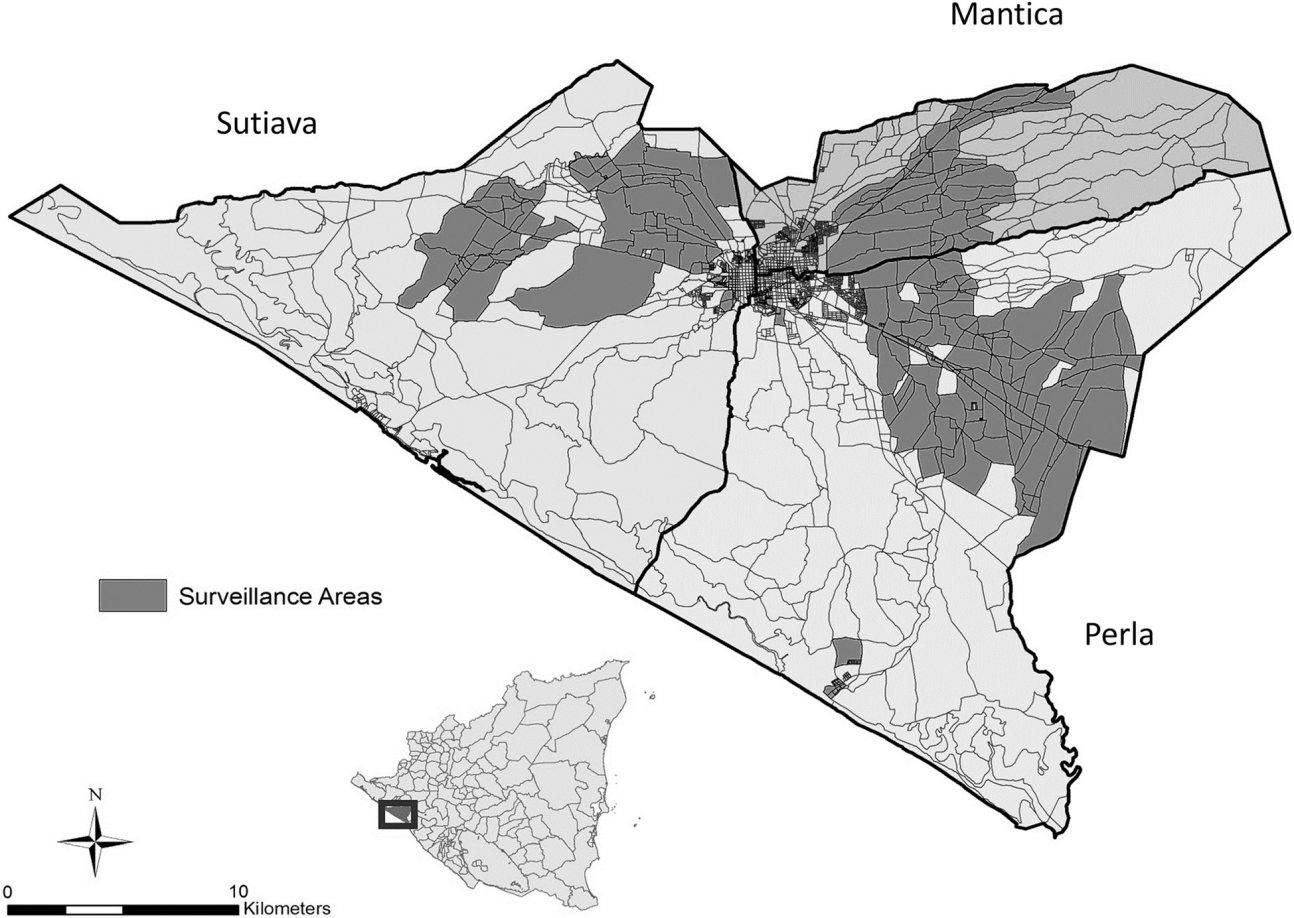
**León municipality in Nicaragua and map of the León health and demographic surveillance system clusters.** The small map in the bottom left corner of the figure shows the location of León municipality within the country of Nicaragua. The municipality of León has an area of 820 km^2^, making it the second largest city in Nicaragua [8]. The dark grey and black areas in the larger map represent the clusters that form the León Health and Demographic Surveillance System. Cluster sampling was conducted in each territory of the municipality of León: Perla, Sutiava, and Mantica. All three territories include both rural and urban areas.

In 1993, the León Health and Demographic Surveillance System (Figure 1: LHDSS) was established by randomly selecting 50 out of 208 pre-defined geographical clusters, with probability proportional to the number of inhabitants in each cluster. The system thus captures a representative sample of León residents, amounting to approximately 55,000 people in 11,000 households [8]. From this surveillance population, we randomly selected 3000 individuals aged 18–70. Written informed consent was obtained from all participants. Institutional Review Boards of University of North Carolina and the National Autonomous University of Nicaragua in León (UNAN-León) approved all study activities.

### Ascertainment of covariate and outcome data

Participants were consented and interviewed in their homes. A study nurse drew a blood sample, which was stored on ice and transported to the biochemistry laboratory at the medical campus of UNAN-León. Serum samples were analyzed for creatinine levels within 24 hours of collection. Study visits were conducted between April and September 2010 and October and November 2011 by trained staff at UNAN-León, and biological specimens were analyzed by qualified laboratory technicians.

### Demographic and exposure definitions

Demographic indicators were sex, age, education level, household poverty, zone (urban vs. rural), neighborhood (Sutiava, Mantica or Perla) and source of drinking water. Drinking water source was categorized into four types: community source, indoor plumbing, own well, and other (includes people who obtain water from a river or stream, a neighbor, or purchased water in barrels or jugs). As participants could indicate more than one water source, these categories are not mutually exclusive. A household-level relative poverty indicator, based on the unsatisfied basic needs index score [9], was calculated using data collected through the demographic surveillance system (Additional file 1: Figure S1). This poverty indicator has been validated in the Nicaraguan setting [10]. Poverty data were unavailable for 247 participants from the Sutiava neighborhood. Education level was categorized into four levels: no schooling or illiterate, primary school, secondary school and university/professional school. Diabetes and hypertension diagnoses were self-reported (Yes/No). Occupational and behavioral exposures evaluated included: number of years worked in agriculture, number of days per week consumed *lija*, number of cigarettes smoked per day; number of glasses of water consumed per day (ranked quartiles). To show the full range of the data, years of agricultural work was categorized into a 5-level variable (none, >0-4, 5–9, 10–14, and 15+); however, because the number of females in several of those categories was small, we also collapsed categories to create a 3-level categorical variable for agricultural work (<1, 1–9, and 10+) in sex-stratified analyses.

### Outcome definition

Serum creatinine data were used to estimate glomerular filtration rate (GFR). The abbreviated modification of diet in renal disease (MDRD) equation for non-African Americans [11] was chosen over the CKD-EPI equation to facilitate comparison of results between our study and other studies in the region. Markers of kidney damage and urinary abnormalities were not available; therefore the primary outcome of CKD was defined as an estimated GFR (eGFR) of <60 ml/min/1.73 m^2^, calculated using a single measurement of serum creatinine. Descriptive analyses were carried out in three categories of kidney function: eGFR <60 ml/min/1.73 m^2^ (presumed CKD), 60- < 90 ml/min/1.73 m^2^ (decreased GFR), and ≥90 ml/min/1.73 m^2^ (presumed healthy). A dichotomous eGFR variable (<60 vs. ≥ 60 ml/min/1.73 m^2^) was used in analyses of factors associated with CKD. Values of eGFR above 150 ml/min/1.73 m^2^ were truncated at 150 as levels above this are not considered an accurate measure of hyperfiltration [12].

The measurement of serum creatinine was calibrated in anticipation of study commencement. One hundred samples across a wide range of serum creatinine levels were collected at UNC, divided into two aliquots and frozen at–70 degrees Celsius. One aliquot of each sample was evaluated by the UNC Hospital Core Laboratory and the other by the UNAN laboratory. Values derived from Passing-Bablok linear regression analyses were used to convert the León-derived creatinine values to UNC-standardized creatinine values [13]. Because the UNC laboratory uses the isotope dilution mass spectrometry (IDMS) reference method to calibrate creatinine values, creatinine measurements in our study have been appropriately standardized for use with the MDRD and CKD-EPI methods [11]. Participants’ blood samples were analyzed using the modified Jaffe method to measure serum creatinine levels [14].

### Statistical analyses

#### Descriptive analyses

Prevalence proportions of three eGFR categories (<60, 60- < 90, ≥90) and 95% confidence intervals were calculated for measured covariates. Observations missing eGFR data were not included in prevalence calculations. Statistical tests were conducted to assess homogeneity of prevalence estimates across the three levels of eGFR: analysis of variance (ANOVA) for age and Mantel-Haenszel chi-square test for categorical variables. A p-value of <0.05 indicated a rejection of the null hypothesis of homogeneity across exposure and outcome groups.

CKD Prevalence estimates were directly standardized to the LHDSS population by age, sex, education, poverty, neighborhood, and geographic zone. Age-standardized prevalence proportions were calculated using 2005 León Census [15] as the reference population.

#### Logistic regression

Variables significantly correlated with CKD in descriptive analyses were carried forward for review in factor analyses. A pairwise correlation matrix was evaluated with the plan of not controlling for both measures in statistical modeling when any two predictor variables attained correlations of 0.70 or greater. Bivariate logistic regression models were first fitted to evaluate the crude association between these factors and dichotomous CKD (eGFR < 60 vs. ≥60). All multivariable logistic regression models were adjusted for potential confounders including age, sex, diabetes, and high blood pressure. In addition, we estimated adjusted prevalence odds ratios stratified by sex.

#### Comparison of MDRD and CKD-EPI equation

The MDRD study equation is reasonably accurate at eGFRs of less than 60 mL/min per 1.73 m^2^; however, its major limitations are imprecision and systematic underestimation of measured GFR at higher values, leading to overestimation of CKD prevalence. The CKD-EPI equation has less bias at high eGFRs and is more accurate for predicting adverse outcomes than is the MDRD equation [16]. To assess the extent of bias in CKD prevalence estimates, we calculated prevalence of eGFR < 60 overall, by sex, by age category, and by age and sex category using the CKD-EPI equation. For prevalence proportions <1%, the Wilson score interval was used to calculate 95% confidence intervals [17]; otherwise the standard Wald method was used. McNemar’s tests for difference in proportions within subjects were performed to assess homogeneity of the CKD definition using the CKD-EPI and MDRD equations. Because CKD-EPI estimates are always lower than MDRD estimates, we used a one-sided p-value (α = 0.1). Using a Bonferroni correction for multiple comparisons, our critical value for significance was 0.1/18 = .0056.

#### Sensitivity analysis: Cutpoint for CKD case definition

EGFR < 60 ml/min/1.73 m^2^ is widely accepted as an appropriate cutpoint for determining CKD; however, the potential for error exists with a single measure of serum creatinine to estimate eGFR. To assess the impact of potential outcome misclassification, we conducted sensitivity analyses comparing adjusted prevalence odds ratios using two additional eGFR cut-point criteria: <90 versus ≥90 ml/min/1.73 m^2^ and <60 versus >90 (with eGFR from 60 to 90 excluded).

Analyses were performed with SAS version 9.2 (Cary, NC, USA). All significance testing was two-sided unless otherwise stated.

## Results

Of the 3000 individuals initially selected to participate in this study, 2493 completed questionnaires, with 91% (n = 2275) also providing blood samples for analysis of serum creatinine. Seven percent of those approached were not available for interview, the majority of whom had migrated, and less than 10% refused. A number of participant demographic characteristics were different compared to those of the surveillance system (Table 1), which is why overall and sex-specific prevalence estimates were standardized to population-representative surveillance and census population data (see Table 2).

**Table 1:** **Comparison of León health and demographic surveillance system and participant data**

**Indicator**	León health and demographic surveillance system*		**Participants**		
	**N**	**%**	**N** ^**§**^	**%**	**p-value** ^**‡**^
**Total**	32,591		2493		
Geographic zone					
Urban	23,392	71.8	1585	63.7	<.0001
Rural	9,199	28.2	903	36.3	
Neighborhood					
Perla	13,650	41.9	975	39.8	0.0401
Mantica	9,240	28.4	668	27.2	0.2399
Sutiava	9,701	29.8	809	33.0	0.0008
Education					
No schooling	3,649	11.2	251	10.2	0.127
Primary	11,811	36.2	1020	41.6	<.0001
Secondary	12,035	36.9	728	29.7	<.0001
University/Professional	4,933	15.1	451	18.4	<.0001
Poverty index**					
Not poor	21,387	65.6	1207	54.7	<.0001
Poor	10,263	31.5	935	42.4	<.0001
Extremely poor	941	2.9	63	2.9	0.9347
Age					
18-29	13,594	41.7	595	24.3	<.0001
30-39	7,154	22.0	781	31.8	<.0001
40-49	5,727	17.6	489	19.9	0.0031
50-59	3,736	11.5	361	14.7	<.0001
60-70	2,380	7.3	227	9.3	0.0004
Sex					
Male	15,214	46.7	1054	42.4	<.0001
Female	17,377	53.3	1434	57.6	

*Surveillance system data among residents aged 18–70.

^§^Numbers within each variable do not sum to the total because of missing values and percentages were calculated based on available data for each variable. Percent missing GFR data for each variable is presented in Table 3.

^‡^Mantel-Haenszel Chi-Square test for homogeneity of proportions.

**Missing poverty data for participants from rural Sutiava (N = 247).

**Table 2:** **CKD prevalence (%): study results and standardized results**

**Overall**				
Study participants	9.1			
Standardized to LHDSS demographic data by:				
Sex	9.5			
Zone	8.6			
Education	8.8			
Area (Neighborhood)	9.0			
Age	7.6			
Poverty	7.7			
**Age-standardized sex-specific prevalence**	**Male**		**Female**	
Study participants	13.8		5.8	
Using age distribution by sex (2005 León Census)	12.0		4.8	
**Age-standardized sex-specific prevalence by geographic zone**	**Urban Male**	**Rural Male**	**Urban Female**	**Rural Female**
Study Participants	9.8	17.8	5.2	7.5
Using zone and age distributions by sex (2005 León Census)	8.1	16.1	4.5	6.0

### Standardized prevalence

Overall standardized prevalence of eGFR < 60 ranged from 7.6% (age-standardized) to 9.5% (sex-standardized), compared to a study prevalence of 9.1% (Table 2). Age-standardized prevalence proportions for males and females were lower than crude prevalence proportions estimated using study data, overall and by zone. The pattern of higher prevalence among males and among rural residents was maintained.

### Characteristics of participants

More women than men participated in this study (58%), participant ages were skewed toward younger ages, and 64% lived in urban areas reflecting the predominance of these characteristics in the León population (Table 3). The majority of participants had some education (primary 42%; secondary 30%; university or professional school 18%). Much of the study population were defined as living in poverty, with 42% considered poor and an additional 3% considered extremely poor. Most participants had access to water in the home, with 86% having indoor plumbing, while 19% of participants obtained water from a personal well, 9% from a community source, and 4% from other sources (percentages do not add up to 100 because categories are not mutually exclusive).

**Table 3:** **Stratum-specific prevalence of chronic kidney disease, León, Nicaragua by demographic characteristics and potential risk factors**

	**N (%)** ^**†**^	**Percent missing**	**Stratum-specific prevalence (95% CI)** ^*^			**p-value** ^**§**^
			**0 < =GFR < 60**	**60 < =GFR < 90**	**GFR= > 90**	
			***N = 208***	***N = 493***	***N = 1574***	
		**GFR data**				
		***N = 218***				
Mean eGFR ± SD^‡^	2493	8.7	39 ± 15	78 ± 8	121 ± 20	
**Demographic Characteristics**						
Sex						
Female	1434 (58)	7.7	5.8 (4.6, 7.1)	19.9 (17.8, 22.1)	74.2 (71.9, 76.6)	<.0001
Male	1054 (42)	9.8	13.8 (11.6, 16)	24.1 (21.4, 26.8)	62.1 (59.1, 65.2)	
Age (continuous)						
	2493(100)	1.6	51.4 ± 11.8	45.3 ± 11.7	36.2 ± 11.4	
Age (categorical)						
18-29	595 (24)	7.9	1.6 (0.6, 2.7)	9.1 (6.7, 11.5)	89.2 (86.6, 91.8)	
30-39	781 (32)	6.8	3.8 (2.4, 5.2)	14.6 (12, 17.1)	81.6 (78.8, 84.4)	<.0001
40-49	489 (20)	8.2	8.9 (6.3, 11.5)	34.1 (29.7, 38.5)	57.0 (52.4, 61.6)	
50-59	361 (15)	6.1	20.1 (15.8, 24.3)	35.1 (30, 40.2)	44.8 (39.5, 50.1)	
60-70	227 (9)	7.0	29.9 (23.7, 36)	30.8 (24.6, 37)	39.3 (32.7, 45.9)	
Geographic Zone						
Urban	1585 (64)	8.8	6.7 (5.4, 8.0)	20.8 (18.7, 22.9)	72.5 (70.2, 74.8)	
Rural	903 (36)	8.1	13.4 (11.1, 15.7)	23.1 (20.3, 26)	63.5 (60.2, 66.8)	<.0001
Neighborhood						
Mantica	668 (27)	8.1	6.4 (4.4, 8.3)	20.2 (17, 23.4)	73.5 (70.0, 76.9)	
Perla	975 (40)	8.5	9.0 (7.1, 10.8)	26.2 (23.3, 29.1)	64.8 (61.7, 67.9)	0.0039
Sutiava	809 (33)	5.1	11.6 (9.3, 13.9)	17.6 (14.9, 20.3)	70.8 (67.6, 74.0)	
Education						
No schooling	251 (10)	5.2	22.7 (17.4, 28)	21.8 (16.6, 27.1)	55.5 (49.1, 61.8)	
Primary	1020 (42)	7.5	12.0 (9.9, 14.1)	25.0 (22.3, 27.8)	63.0 (59.9, 66.1)	
Secondary	728 (30)	7.6	3.7 (2.3, 5.1)	18.3 (15.4, 21.2)	78.0 (74.9, 81.1)	<.0001
University/Professional	451 (18)	7.3	3.6 (1.8, 5.4)	19.4 (15.6, 23.2)	77.0 (73.0, 81.1)	
Poverty Index^**^						
Not Poor	1207(55)	7.5	7.5 (6.0, 9.0)	24 (21.6, 26.4)	68.5 (65.8, 71.1)	0.3308
Poor	935(42)	7.7	7.9 (6.2, 9.6)	18.7 (16.2, 21.2)	73.5 (70.6, 76.3)	
Extremely poor	63(3)	14.3	9.3 (2.1, 16.4)	27.8 (16.7, 38.8)	63.0 (51.0, 74.9)	
Water Source^††^						
Indoor Plumbing	1894 (86)	5.4	5.6 (4.6, 6.5)	17 (15.4, 18.5)	57.2 (55.2, 59.3)	
Community Source	198 (9)	0.5	1.1 (0.7, 1.6)	1.8 (1.2, 2.3)	5.5 (4.6, 6.5)	
Own well	414 (19)	1.5	2.9 (2.2, 3.7)	4.0 (3.2, 4.8)	10.1 (8.9, 11.4)	
Other	82 (4)	0.3	0.5 (0.2, 0.7)	0.6 (0.3, 1.0)	2.3 (1.7, 2.9)	
**Potential Risk Factors**						
Number of times drank unregulated alcohol (lija)						
None	2332 (95)	7.2	8.5 (7.4, 9.7)	21.8 (20, 23.5)	69.7 (67.7, 71.6)	0.0004
≥1 per week	121 (5)	8.3	20.7 (13.2, 28.3)	19.8 (12.4, 27.2)	59.5 (50.3, 68.6)	
Number of cigarettes smoked						
None	2131 (87)	7.3	8.9 (7.6, 10.1)	21 (19.2, 22.8)	70.2 (68.2, 72.2)	0.0166
≥1 per day	322 (13)	6.8	11.0 (7.5, 14.5)	26.3 (21.3, 31.3)	62.7 (57.2, 68.1)	
Self-reported diabetes						
No	2342 (95)	7.3	8.4 (7.3, 9.6)	21 (19.3, 22.7)	70.6 (68.7, 72.5)	<.0001
Yes	111 (5)	7.2	24.3 (16, 32.6)	35.9 (26.7, 45.2)	39.8 (30.4, 49.3)	
Self-reported high blood pressure						
No	2127 (87)	7.3	7.4 (6.2, 8.5)	20.6 (18.8, 22.4)	72.0 (70.1, 74.0)	<.0001
Yes	326 (13)	6.7	20.7 (16.2, 25.3)	28.6 (23.5, 33.7)	50.7 (45, 56.3)	
Number of years worked in agriculture						
never	1365 (56)	7.0	4.5 (3.4, 5.6)	18.4 (16.2, 20.5)	77.1 (74.8, 79.5)	<.0001
>0 to 4	325 (13)	8.6	6.7 (3.9, 9.6)	24.6 (19.7, 29.5)	68.7 (63.4, 74.0)	
5-9	201 (8)	7.0	9.6 (5.4, 13.9)	27.8 (21.4, 34.2)	62.6 (55.6, 69.5)	
10-14	187 (8)	4.3	15.6 (10.3, 21)	22.9 (16.7, 29.1)	61.5 (54.3, 68.6)	
≥15	375 (15)	8.5	24.8 (20.2, 29.4)	27.4 (22.7, 32.1)	47.8 (42.5, 53.1)	
Daily water consumption^‡‡^						
0-4	699 (29)	6.9	5.2 (3.5, 6.9)	17.8 (14.9, 20.8)	77 (73.7, 80.2)	<.0001
5-8	808 (33)	7.3	7.9 (5.9, 9.8)	20.8 (17.9, 23.7)	71.3 (68.1, 74.5)	
9-12	505 (21)	7.9	12.5 (9.5, 15.5)	23.2 (19.4, 27.1)	64.3 (59.9, 68.7)	
13-52	439 (18)	7.1	13.7 (10.4, 17.1)	27.7 (23.4, 32.0)	58.6 (53.8, 63.4)	

*Prevalence was calculated as the number of participants in each eGFR by covariate strata, divided by the total number of participants in the relevant covariate strata. For example, the prevalence of females with eGFR < 60 was calculated as the number of females with eGFR < 60 divided by the total number of females in the study (i.e. 77/1324 = 5.8%).

^†^Percentages may not add up to 100 due to rounding.

^‡^Calculated using the MDRD method [11] eGFR variable truncated at 150.

^§^p-value for chi-square test of homogeneity.

**Household-level relative poverty indicator calculated using data from health surveillance system that takes into consideration education level, occupation of each household member, water source, material predominantly used in home construction, and toilet facilities (see Additional file 1: Figure S1).

^††^Percentages do not add up to 100 because categories are not mutually exclusive.

^‡‡^Quartiles of ranked average number of glasses of water per day.

Few participants reported having a prior diabetes diagnosis (5%), and only 13% reported a prior diagnosis of high blood pressure. Thirteen percent of participants reported smoking ≥1 cigarette(s) per day, and 5% percent of participants reported drinking lija ≥1 time(s) per week, though only 2 of those were women. History of agricultural work was more common among men (64%) compared to women (30%) (data not shown). Forty-four percent of participants reported a history of agricultural work; of those, more than half reported working in agriculture for ≥10 years. The majority of participants (62%) reported drinking eight or fewer glasses of water per day.

### Prevalence of estimated Glomerular Filtration Rate (eGFR) <60

Prevalence of CKD among men was more than double the prevalence among women (13.8% vs. 5.8%: Table 3). Prevalence of CKD was significantly greater among participants in older age groups (20.1% for those aged 50–59 and 29.9% for those aged 60–70). Mean age was highest for those with eGFR < 60 (51 yrs ± 12) compared to those with 60 ≤ eGFR < 90 (45 yrs ± 12) and those with an eGFR ≥ 90 (36 yrs ± 11). Rural areas had higher prevalence (13.4%) than urban areas (6.7%), with the highest prevalence observed in Sutiava (11.6%), and no difference was seen across three levels of household poverty.

Prevalence of CKD was 24.3% among participants with self-reported diabetes and 8.4% among those without a diabetes diagnosis. CKD prevalence was 20.7% among participants who self-reported high blood pressure, and 7.4% among those who did not. CKD prevalence was significantly higher among participants who reported drinking *lija* one or more times per week (20.7%). No significant difference was seen between smokers and non-smokers. Prevalence of CKD increased with increasing years of agricultural work and higher levels of water consumption per day. Prevalence was also markedly higher among those with no schooling compared to those with primary, secondary, or university/professional education.

### Comparison of MDRD and CKD-EPI equations

Prevalence of CKD in León was attenuated in all strata when GFR was estimated using the CKD-EPI equation instead of the MDRD equation (Table 4). Prevalence estimates using the CKD-EPI equation were significantly lower for the overall study population and within all but the youngest two age groups. Among females, CKD-EPI estimates were significantly lower overall and in the two oldest age categories. For males, only the total prevalence estimate was significantly lower using CKD-EPI. Prevalence of CKD was higher among males for all age groups, regardless of equation used.

**Table 4:** **CKD Prevalence (%) and 95% confidence intervals by age and sex: MDRD vs. CKD-EPI**

	**Total**					**Males**					**Females**				
	MDRD*		**CKD-EPI** ^**†**^			**MDRD**		**CKD-EPI**			**MDRD**		**CKD-EPI**		
**Age**	**N**	**Prevalence (95% CI)**	**N**	**Prevalence (95% CI)**	**P** ^**‡**^	**N**	**Prevalence (95% CI)**	**N**	**Prevalence (95% CI)**	**p**	**N**	**Prevalence (95% CI)**	**N**	**Prevalence (95% CI)**	**p**
18-29	9	1.6 (0.5, 2.7)	6	1.1 (0.2, 2.0)	0.3§	8	3.8 (1.2, 6.4)	5	2.4 (0.3, 4.5)	0.3^§^	1	0.3 (0.1, 1.7)	1	0.3 (0.1, 1.7)	--
30-39	28	3.8 (2.4, 5.2)	22	3 (1.8, 4.2)	0.01	23	6.9 (4.2, 9.6)	19	5.7 (3.2, 8.2)	0.1^§^	5	1.3 (0.2, 2.4)	3	0.8 (0.3, 2.2)	0.5^§^
40-49	40	8.9 (6.3, 11.5)	32	7.1 (4.7, 9.5)	0.005	28	15.4 (10.2, 20.6)	26	14.3 (9.2, 19.4)	0.5^§^	12	4.5 (2.0, 7.0)	6	2.2 (0.4, 4.0)	0.01
50-59	68	20.1 (15.8, 24.4)	57	16.8 (12.8, 20.8)	0.0009	46	30.9 (23.5, 38.3)	43	28.9 (21.6, 36.2)	0.3^§^	22	11.6 (7.0, 16.2)	14	7.4 (3.7, 11.1)	0.005
60-70	48	29.9 (23.7, 36.1)	53	25.1 (19.2, 31.0)	0.002	26	34.7 (23.9, 45.5)	25	33.3 (22.6, 44)	1.0^§^	37	27.2 (19.7, 34.7)	28	20.6 (13.8, 27.4)	0.003
Total	208	9.1 (7.9, 10.3)	170	7.5 (6.4, 8.6)	<0.0001	131	13.8 (11.6, 16.0)	118	12.4 (10.3, 14.5)	0.0003	77	5.8 (4.5, 7.1)	52	3.9 (2.9, 4.9)	<0.0001

*MDRD: Modification of Diet in Renal Disease equation for estimating glomerular filtration rate.

^†^CKD-EPI: Chronic Kidney Disease Epidemiology Collaboration equation for estimating glomerular filtration rate.

^‡^p-value for Mcnemar’s test for paired data.

^§^Mcnemar’s exact test used for sparse data (i.e. when value for discordant pair MDRD=’yes’ and CKD-EPI=”no” is less than 5).

### Analysis of risk factors for CKD

In bivariate analyses (Table 5), older age, male sex, rural zone, lower education level, self-reported diagnosis of high blood pressure, self-reported diagnosis of diabetes, more years of agricultural work, lija consumption, and higher levels of average daily water consumption were significantly associated with CKD. All pair-wise correlations of variables studied were between zero and ±0.4 with only three exceptions, years in agriculture and zone (ρ = 0.59), zone and education (ρ = −0.45) and education and years in agriculture (ρ = −0.45), none of which reached our a priori correlation cut-point of 0.70 for modeling. In multivariable models controlling for age, sex, blood pressure and diabetes, all variables except for diabetes remained statistically significantly associated with prevalence of CKD at the alpha = 0.05 level. Men had an adjusted odds of CKD more than 3 times that of women (POR = 3.47; 95% CI: 2.50, 4.80), and this estimate remained statistically significant even when we further controlled for other factors associated with CKD prevalence and moderately correlated with sex, including zone (rural vs. urban), lija consumption, years of agricultural work, and water consumption (POR = 2.17; 95% CI: 1.49, 3.15, data not shown in Table 5). Living in a rural zone was associated with significantly increased prevalence of CKD (POR = 2.10; 95% CI: 1.53, 2.89). The prevalence odds ratio for those reporting a diagnosis of high blood pressure was 2.07 (95% CI: 1.43, 3.02), compared to those who did not report a diagnosis.

**Table 5:** **Associations between chronic kidney disease and potential risk factors, León, Nicaragua**

	**<60 eGFR** ^**§ **^ **N (%)** ^**†**^	**≥60 eGFR N (%)** ^**†**^	**Unadjusted POR (95% CI)**	**p-value**	Adjusted POR (95% CI)*	**p-value**
Sex						
Female	77 (37)	1247 (60)	*referent*	--	*referent*	
Male	131 (63)	820 (40)	2.59 (1.93, 3.47)	<.0001	3.47 (2.50, 4.80)	<.0001
Age (categorical)						
18-29	9 (4)	539 (26)	*Referent*		*referent*	
30-39	28 (13)	700 (34)	2.39 (1.12, 5.11)	0.0242	2.10 (0.98, 4.51)	0.0567
40-49	40 (19)	409 (20)	5.85 (2.81, 12.20)	<.0001	5.10 (2.43, 10.72)	<.0001
50-59	68 (33)	271 (13)	15.02 (7.38, 30.55)	<.0001	11.96 (5.79, 24.71)	<.0001
60-70	63 (30)	148 (7)	25.48 (12.38, 52.42)	<.0001	23.3 (11.12, 48.83)	<.0001
Geographic Zone						
Urban	97 (47)	1348 (65)	*referent*		*referent*	
Rural	111 (53)	719 (35)	2.15 (1.61, 2.86)	<.0001	2.10 (1.53, 2.89)	<.0001
Neighborhood						
Mantica	39 (19)	575 (28)	*referent*			
Perla	80 (38)	812 (39)	1.45 (0.98, 2.16)	0.0656	1.67 (1.09, 2.56)	0.0194
Sutiava	89 (43)	679 (33)	1.93 (1.31, 2.86)	0.001	1.95 (1.27, 2.97)	0.0021
Self-reported diabetes						
No	183 (88)	1989 (96)	*referent*		*referent*	
Yes	25 (12)	78 (4)	3.48 (2.17, 5.60)	<.0001	1.37 (0.81, 2.34)	0.2425
Self-reported high blood pressure						
No	145 (70)	1826 (88)	*referent*		*referent*	
Yes	63 (30)	241 (12)	3.29 (2.38, 4.56)	<.0001	2.07 (1.43, 3.02)	0.0001
Education						
University and Professional	15 (7)	403 (20)	*referent*		*referent*	
Secondary	25 (12)	648 (31)	1.04 (0.54, 1.99)	0.9141	1.13 (0.57, 2.24)	0.7364
Primary	113 (55)	830 (40)	3.66 (2.11, 6.35)	<.0001	2.44 (1.35, 4.41)	0.003
No schooling	54 (26)	184 (9)	7.88 (4.34, 14.34)	<.0001	4.24 (2.21, 8.14)	<.0001
Number of years worked in agriculture						
None	57 (27)	1212 (59)	*referent*		*referent*	
>0-4	20 (10)	277 (13)	1.54 (0.91, 2.6)	0.1101	1.39 (0.80, 2.43)	0.24
5-9	18 (9)	169 (8)	2.26 (1.30, 3.94)	0.0038	1.70 (0.94, 3.06)	0.0773
10-14	28 (13)	151 (7)	3.94 (2.43, 6.39)	<.0001	2.55 (1.50, 4.32)	0.0005
15+	85 (41)	258 (12)	7.01 (4.88, 10.06)	<.0001	2.91 (1.94, 4.38)	<.0001
Number of times drank unregulated alcohol (lija)						
None	185 (89)	1979 (96)	*referent*		*referent*	
≥1 per week	23 (11)	88 (4)	2.80 (1.73, 4.53)	<.0001	1.95 (1.13, 3.38)	0.0164
Number of cigarettes smoked						
None	175 (84)	1800 (87)	*referent*		*referent*	
≥1 per day	33 (16)	267 (13)	1.27 (0.86, 1.88)	0.2317	0.86 (0.56, 1.35)	0.5199
Daily water consumption^‡^						
0-4	34 (16)	617 (30)	*referent*		*referent*	
5-8	59 (29)	690 (33)	1.55 (1.00, 2.40)	0.0482	0.98 (0.61, 1.56)	0.9201
9-12	58 (28)	407 (20)	2.59 (1.66, 4.02)	<.0001	1.62 (1.00, 2.63)	0.0488
13-52	56 (27)	352 (17)	2.89 (1.85, 4.51)	<.0001	1.80 (1.08, 2.98)	0.0231

^†^Percentages may not add up to 100 due to rounding.

*Logistic regression model adjusted for sex, age (indicator-coded categorical variable), self-reported high blood pressure diagnosis, and self-reported diabetes diagnosis.

^§^Calculated using the MDRD method [11] eGFR variable truncated at 150.

^‡^Quartiles of ranked average number of glasses of water per day.

**Figure 2:**
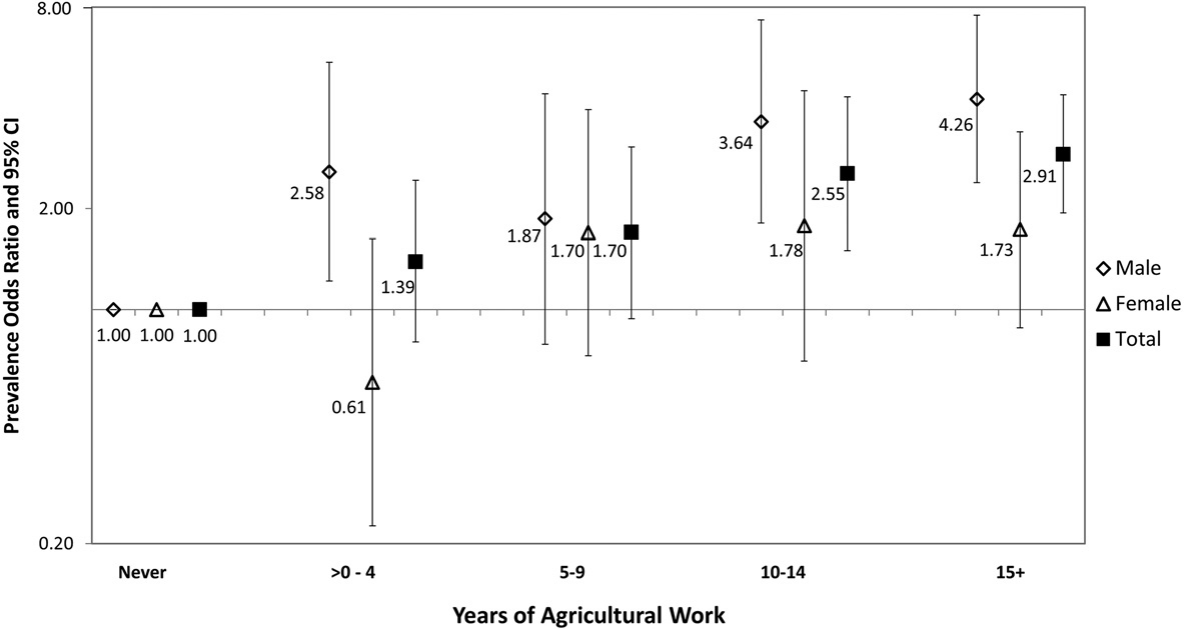
**This figure shows the prevalence odds ratios and 95% confidence intervals for the association with years of agricultural work.** Those who reported no years of agricultural work form the referent group. Estimates among males (diamond), females (triangle), and overall (square) increase with increasing numbers of years of agricultural work, but estimates among males are consistently higher than estimates among females.

Those who reported drinking *lija* had a higher odds of CKD compared to those who did not (POR = 1.95; 95% CI: 1.13, 3.38). More years of work in agriculture was associated with a significantly higher odds of CKD compared to never working in agriculture (Figure 2), with prevalence odds ratios of 2.55 (95% CI: 1.50, 4.32) and 2.91 (95% CI: 1.94, 4.38) for those who reported working in agriculture for 10–14 years and ≥15 years, respectively. When limited to men, models revealed that years of agriculture remained highly important and conferred an increasing odds of CKD with increasing years of agricultural work as in the overall study data (Figure 2). Among women, the odds of CKD was significantly elevated among those who worked in agriculture for ten or more years, though estimates were less precise (Additional file 2: Table S1). Of all models evaluated, the one limited to women showed the only impact of a self-reported history of diabetes with CKD (OR = 2.52, 95% CI: 1.23, 5.16 vs. model limited to men: OR = 0.78, 95% CI: 0.34, 1.79), adjusting for age, high blood pressure and years of agricultural work (data not shown in Additional file 2: Table S1).

Participants who drank 13–52 glasses of water per day had a higher odds of CKD compared to those who drank 0–4 glasses of water per day (POR = 1.80; CI: 1.08, 2.98). Prevalence odds ratios for CKD increased with decreasing educational achievement. Odds of CKD for participants with no schooling were 4.24 times the odds of CKD for participants who attended university or professional school (95% CI: 2.21, 8.14).

### Sensitivity analysis results

The magnitude of adjusted prevalence odds ratios and confidence limit ratios were similar when primary outcome results were compared to results using eGFR <60 vs. >90. Compared to the primary outcome categorization, effect estimates using an outcome categorization of eGFR <90 vs. ≥ 90 were generally muted. A few associations that were not significant using the primary outcome categorization became significant when using the eGFR <90 vs. ≥ 90 categorization, including self-reported diabetes (POR = 1.65; 95% CI: 1.05, 2.59), 30–39 year olds vs. 18–29 year olds (POR = 1.74; 95% CI: 1.25, 2.43), >0-4 years of agricultural work vs. none (POR = 1.39; 95% CI: 1.03, 1.88), and 5–9 years of agricultural work vs. none (POR = 1.65; 95% CI: 1.23, 2.21). Inspection of the confidence limits revealed increased precision rather than substantial inflation of these estimates.

## Discussion

In this large, population-based study we found an overall CKD prevalence of 9.1% using the MDRD equation and 7.5% using CKD-EPI. These estimates are comparable to those found in regional studies, and validate the growing body of research indicating a CKD epidemic in the Western Pacific coastal region of Central America [1,2,4-7,18]. Our study improves upon existing research in several ways. First, prior research used small non-population-based samples from mainly rural areas. The use of random sampling methodology in this study improves generalizability of study results. Second, León’s geographic and occupational diversity and existing demographic surveillance system make it an ideal location for assessing CKD prevalence across population characteristics. Third, the sample size in this study is over twice that of all prior studies in Nicaragua and, therefore, permits far more precise quantification of the associations between CKD prevalence and a variety of demographic and occupational factors, providing direction for future study.

The high prevalence among men in this study was striking. Whereas a higher prevalence of CKD among women is reported in both developed and developing nations [19], prevalence of CKD is reportedly higher among males in the Pacific coastal region of Central America, ranging from 14-26% [1-4,7]. Our study found comparably high prevalence of eGFR < 60 among men (13.8%), more than twice that of women, and higher prevalence among males remained significant after adjustment for all other potential risk factors for CKD. CKD was present in women, but it was confined mainly to the older age groups. Direct standardization of study prevalence proportions using demographic data from the LHDSS and León Census revealed a general overestimation of prevalence, but prevalence estimates remained remarkably high, particularly among males living in rural areas (age-adjusted prevalence: 16.1%). A study by O’Donnell et al. estimated an 11% prevalence of moderately decreased kidney function (eGFR 30–59) and a 9% prevalence of severely decreased kidney function (eGFR < 30) among Nicaraguan men, while our study found CKD prevalence proportions of 7.9% and 3.6% among respective eGFR categories. By comparison, in the United States, where prevalence of diabetes and hypertension among the adult population are 10% and 40% respectively [20], eGFR categories among males were 6% for eGFR 30–59 and 0.5 for eGFR < 30 [21].

As expected, CKD was associated with older age in our study, with prevalence estimates ranging from 1.6% in the youngest age category to 29.9% in the oldest age category. Yet, prevalence estimates among male study participants were significantly greater than estimates for females in all age categories. Our results mirror those of other studies, which show an elevated CKD prevalence among young adult males in the Pacific region of Central America [1-4,7].

High prevalence in this region has not been sufficiently explained by traditional biological risk factors. In our study, self-reported high blood pressure was a significant risk factor, but high blood pressure is a precursor to and an effect of CKD, making interpretation of a significant association between these two conditions problematic. Self-reported diabetes was a significant risk factor among females but not males, a finding consistent with results from a similar study in the region [4]. The lack of association between CKD and diabetes among males in this study supports a hypothesis of a non-conventional CKD etiology in this region. This observation is strengthened by a recent publication which reported a new and unique renal disease morphology among El Salvadorian plantation workers diagnosed with chronic kidney disease of unknown origin [22].

Similar to other studies conducted in Pacific coastal Central America [1,2,4,6], we found longer durations of agricultural work to be associated with CKD in multivariate logistic regression models. There are various hypotheses for this finding, including: repeated episodes of subclinical kidney injury due to chronic dehydration from long-term hard labor in conditions known to be conducive to dehydration [23]; consumption of contaminated drinking water at work; chronic exposure to nephrotoxic pesticides; or a combination of all of these factors. The relationship among these factors and with CKD deserves further exploration.

Prior studies have suggested that chronic and severe dehydration may play a role in the development of CKD in affected populations [18]. Our finding of increased water consumption associated with elevated odds of CKD was also observed in a prior study conducted in a nearby region [1]. A significant positive association between daily consumption of large quantities of water and CKD may reflect higher prevalence of CKD among chronically dehydrated participants, an effect of prolonged ingestion of contaminated water, or an indication of a urine concentrating defect, leading to water loss and thus the need for increased water intake. Because accurate classification of water consumption is difficult using data from a single questionnaire, other methods of measuring daily water intake, such as direct observation or daily dietary records, should be employed in future studies.

Though the proportion of participants who reported drinking *lija* was small (5%), the prevalence odds ratio for *lija* consumption vs. none was significant. Sanoff et al. [1] found increasing quartiles of *lija* consumption to be associated with increased odds of CKD. O’Donnell et al. also observed a positive association between *lija* and CKD, although the association was no longer significant after adjusting for age and sex [2]. If a causal relationship between *lija* and CKD truly exists, the mechanism for this effect is unknown. Consumers may experience a direct nephrotoxic effect of chronic low grade methanol exposure due to the unregulated manufacture of *lija.* In 2006, *lija* contamination was responsible for 788 cases of methanol poisoning, including 44 deaths, in western Nicaragua [24]. In the United States, moonshine consumption was found to be associated with nephrosclerosis, interstitial nephritis, and renal insufficiency [25]. Furthermore, because *lija* is commonly mixed and stored in industrial metal containers [1], *lija* drinkers may inadvertently ingest small amounts of lead. Lead has been shown to contribute to nephrotoxicity [26], even at blood lead levels below 5 microg/dl [27]. Lija is now tightly regulated in Nicaragua, such that the design of future studies to investigate its role may be a challenge.

We also found a lack of education to be highly associated with CKD. No other studies in this region have found a distinct association between education level and CKD. In a country with such little wealth, education may be a better indicator of socioeconomic status than household poverty level. However, missing poverty data from the poorest neighborhood, Sutiava, may have biased estimates towards no association between poverty and CKD. Additionally, odds of CKD was elevated in Perla and Sutiava compared to Mantica, though the reason for this is unknown. Future studies should evaluate environmental and structural factors that may be different by neighborhood, such as water contamination and distance to a local clinic.

There are several limitations to our study. First, the cross-sectional nature of our study precludes the establishment of temporality between exposures and the outcome. However, assessing the true magnitude of this health problem in a large random sample of the population is an important first step. Second, due to the exploratory nature of this study and its purpose to inform future research, we were not able to obtain blood pressure measurements, diabetes biomarkers, height and weight, history of transplant, or information on non-steroidal anti-inflammatory drug (NSAID) or prescription medication use. Higher diagnosis rates of hypertension and diabetes among cases vs. non-cases would bias results up and away from the null, whereas we did not observe an association with diabetes in adjusted analyses. Also, our finding of self-reported diabetes prevalence of 5% was comparable to the prevalence of self-reported diabetes (5.3%) in a survey of diabetes and hypertension prevalence in Managua [28], while our finding of 13% self-reported hypertension appears to be lower than that reported in the Managua report (17.6%). Still, the lack of clinical measurements of these conditions is a limitation, and efforts to clinically evaluate these conditions in future studies is necessary. Kidney transplants are extremely rare in Nicaragua [29], and thus would be unlikely to affect our results. Prevalence of NSAID use and association with CKD has varied widely across studies in Nicaragua and El Salvador [1-3,6,7,30,31], but frequent NSAID use among agricultural workers who engage in strenuous manual labor may interact with other environmental factors to increase the risk of developing CKD. This interaction should be explored in future analyses. Third, significant differences in demographic factors among study participants compared to the original surveillance sample may limit the generalizability of study findings. However, prevalence proportions changed minimally when results were standardized to the demographic distribution of the surveillance population and patterns within demographic categories (e.g. high prevalence among males) were maintained. Fourth, the use of a single serum creatinine measurement to estimate GFR may result in some outcome misclassification. Further, the use of an estimating equation that has not been validated in Central American populations may contribute to this misclassification. However, the robustness of our findings is supported by a sensitivity analysis which showed an expected shift in adjusted prevalence odds ratios based on two additional outcome definitions. The weaker effect estimates observed with an outcome categorization of eGFR <90 vs. ≥ 90 were likely a consequence of including non-cases in the case definition. Lastly, in the absence of urinalysis, we were unable characterize renal damage or detect early stages of disease.

## Conclusion

In summary, prevalence of CKD is disturbingly high among adult males aged 59 and younger in León, Nicaragua. Elevated prevalence appears to be concentrated in rural zones and among agricultural workers. Our findings confirm those of other studies in the Pacific coastal regions of Central America, which point to the likely contribution of non-traditional risk factors to this burgeoning regional CKD epidemic. Future research should include detailed assessment of agricultural work history and working conditions, pesticide poisoning and heat stress events, historical and current NSAID use, and measurement of toxins in water and food, infectious diseases associated with renal failure, and use of local herbal medicines. Prior histopathological and clinical research has informed a preliminary definition for Mesoamerican Nephropathy [22,32], but additional studies which incorporate microscopic urinalysis, kidney biopsies, and more precise clinical assessments of co-morbidities are needed to definitively characterize the predominant nephropathology in this region. Understanding the prevalence, risk factors, and biological etiology of CKD in Nicaragua is crucial for enabling the Nicaraguan Ministry of Health (MINSA) and external aid organizations to effectively allocate scant prevention and treatment resources within the health sector.

## Additional files

Additional file 1: Figure S1. Poverty Indicator based on Unsatisfied Basic Needs Index. We used the multi-dimensional Unsatisfied Basic Needs Index [9] to calculate and assign a relative poverty level for study participants. This figure depicts the four basic need areas and the process for assigning a score to each household in the demographic surveillance system. Each household’s score was then assigned to each individual living in the household. Additional file 2: Table S1. Associations between chronic kidney disease and potential risk factors, León, Nicaragua, by sex.

## Abbreviations

ANOVA: Analysis of variance
CKD: Chronic kidney disease
CKD-EPI: Chronic Kidney Disease Epidemiology Collaboration (formula for calculating estimated glomerular filtration rate)
eGFR: Estimated glomerular filtration rate
IDMS: Isotope dilution mass spectrometry
MDRD: Modification of Diet in Renal Disease (formula for calculating estimated glomerular filtration rate)
LHDSS: León Health and Demographic Surveillance System
NSAID: Non-steroidal anti-inflammatory drug
POR: Prevalence odds ratio
UNAN: Spanish acronym for National Autonomous University of Nicaragua
UNC: University of North Carolina–Chapel Hill
