# Supplementary figures and images for: A population-based study of prevalence and risk factors of chronic kidney disease in León, Nicaragua
Source: Can J Kidney Health Dis. 2015 Feb 24;2:6. doi: 10.1186/s40697-015-0041-1 (PMC4414463; doi:10.1186/s40697-015-0041-1)

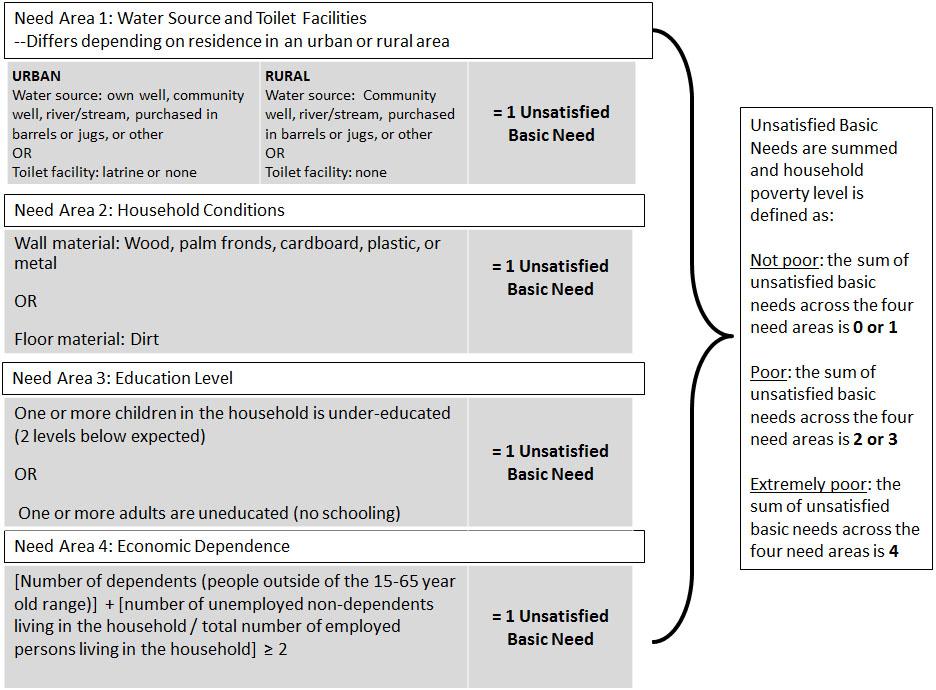

Supplement: Additional file 1: Figure S1. — Poverty Indicator based on Unsatisfied Basic Needs Index. We used the multi-dimensional Unsatisfied Basic Needs Index [9] to calculate and assign a relative poverty level for study participants. This figure depicts the four basic need areas and the process for assigning a score to each household in the demographic surveillance system. Each household’s score was then assigned to each individual living in the household. [file 40697_2015_41_MOESM1_ESM.jpeg]
