# Supplementary material for: A population-based study of prevalence and risk factors of chronic kidney disease in León, Nicaragua
Source: Can J Kidney Health Dis. 2015 Feb 24;2:6. doi: 10.1186/s40697-015-0041-1 (PMC4414463; doi:10.1186/s40697-015-0041-1)
Supplement: Additional file 2: Table S1. — Associations between chronic kidney disease and potential risk factors, León, Nicaragua, by sex. [file 40697_2015_41_MOESM2_ESM.docx]

**Table 5 (Supplement): Associations between chronic kidney disease and potential risk factors, León, Nicaragua, by sex**

|  | **FEMALE** | | | | | | | | | |  | **MALE** | | | | | |
| --- | --- | --- | --- | --- | --- | --- | --- | --- | --- | --- | --- | --- | --- | --- | --- | --- | --- |
|  | **<60 eGFR**§  **N (%)†** | | **≥ 60 eGFR**  **N (%)†** | | **Unadjusted POR**  **(95% CI)** | | **p-value** | | **Adjusted* POR**  **(95% CI)** | **p-value** |  | **<60 eGFR**§  **N (%)†** | **≥ 60 eGFR**  **N (%)†** | **Unadjusted POR (95% CI)** | **p-value** | **Adjusted* POR**  **(95% CI)** | **p-value** |
| Age (categorical) | | |  | |  | |  | |  |  |  |  |  |  |  |  |  |
| 18-29 | 1 (1) | | 335 (27) | | *referent* | |  | | *referent* |  |  | 8 (6) | 204 (25) | *referent* |  | *referent* |  |
| 30-39 | 4 (5) | | 389 (31) | | 4.29 (0.50, 36.93) | | 0.1844 | | 4.10 (0.48, 35.25) | 0.1992 |  | 23 (18) | 310 (38) | 1.89 (0.83, 4.31) | 0.1292 | 1.75 (0.76, 3.99) | 0.186 |
| 40-49 | 12 (16) | | 254 (20) | | 15.76 (2.04, 121.97) | | 0.0083 | | 14.37 (1.85, 111.49) | 0.0108 |  | 27 (21) | 154 (19) | 4.63 (2.06, 10.45) | 0.0002 | 3.91 (1.72, 8.90) | 0.0012 |
| 50-59 | 22 (29) | | 167 (13) | | 43.86 (5.86, 328.10) | | 0.0002 | | 34.32 (4.53, 260.25) | 0.0006 |  | 46 (35) | 103 (13) | 11.39 (5.18, 25.02) | <.0001 | 9.36 (4.20, 20.85) | <.0001 |
| 60-70 | 37 (49) | | 99 (8) | | 125.17 (16.97, 923.67) | | <.0001 | | 100.75 (13.50, 751.70) | <.0001 |  | 26 (20) | 48 (6) | 13.53 (5.77, 31.71) | <.0001 | 11.38 (4.79, 27.04) | <.0001 |
| Geographic Zone | | |  | |  | |  | |  |  |  |  |  |  |  |  |  |
| Urban | 49 (64) | | 915 (74) | | *referent* | |  | | *referent* |  |  | 47 (36) | 431 (53) | *referent* |  | *referent* |  |
| Rural | 27 (36) | | 329 (26) | | 1.49 (0.92, 2.43) | | 0.1043 | | 1.34 (0.79, 2.28) | 0.28 |  | 83 (64) | 388 (47) | 1.99 (1.36, 2.92) | 0.0004 | 2.57 (1.69, 3.93) | <.0001 |
| Neighborhood |  | |  | |  | |  | |  |  |  |  |  |  |  |  |  |
| Mantica | 18 (24) | | 328 (26) | | *referent* | |  | |  |  |  | 21 (16) | 246 (30) | *referent* |  |  |  |
| Perla | 33 (43) | | 519 (42) | | 1.16 (0.64, 2.10) | | 0.618 | | 1.24 (0.65, 2.35) | 0.509 |  | 47 (36) | 293 (36) | 1.88 (1.09, 3.23) | 0.0225 | 2.03 (1.14, 3.61) | 0.0165 |
| Sutiava | 25 (33) | | 397 (32) | | 1.19 (0.64, 2.22) | | 0.5742 | | 1.04 (0.53, 2.02) | 0.9141 |  | 62 (48) | 280 (34) | 2.63 (1.56, 4.43) | 0.0003 | 2.76 (1.58, 4.83) | 0.0004 |
| Self-reported diabetes | | |  | |  | |  | |  |  |  |  |  |  |  |  |  |
| No | 61 (80) | | 1198 (96) | | *referent* | |  | | referent |  |  | 120 (92) | 787 (96) | *referent* |  | referent |  |
| Yes | 15 (20) | | 46 (4) | | 6.32 (3.34, 11.93) | | <.0001 | | 2.48 (1.22, 5.04) | 0.0118 |  | 10 (8) | 32 (4) | 2.04 (0.98, 4.25) | 0.0583 | 0.70 (0.31, 1.57) | 0.3815 |
| Self-reported high blood pressure | | | | |  | |  | |  |  |  |  |  |  |  |  |  |
| No | 47 (62) | 1061 (85) | | | *referent* | |  | | *referent* |  |  | 97 (75) | 761 (93) | *referent* |  | *referent* |  |
| Yes | 29 (38) | 183 (15) | | | 3.51 (2.16, 5.72) | | <.0001 | | 1.28 (0.74, 2.21) | 0.3849 |  | 33 (25) | 58 (7) | 4.61 (2.87, 7.39) | <.0001 | 3.17 (1.88, 5.34) | <.0001 |
| Education |  |  | | |  | |  | |  |  |  |  |  |  |  |  |  |
| University and Professional | 5 (7) | 262 (21) | | | *referent* | |  | | *referent* |  |  | 10 (8) | 141 (17) | *referent* |  | *referent* |  |
| Secondary | 39 (51) | 469 (38) | | | 1.15 (0.38, 3.46) | | 0.8071 | | 1.03 (0.33, 3.23) | 0.9566 |  | 73 (56) | 360 (44) | 0.95 (0.42, 2.16) | 0.906 | 1.09 (0.46, 2.57) | 0.8476 |
| Primary | 9 (12) | 411 (33) | | | 4.35 (1.69, 11.17) | | 0.0023 | | 1.64 (0.59, 4.51) | 0.3424 |  | 16 (12) | 237 (29) | 2.9 0(1.46, 5.77) | 0.0025 | 2.68 (1.29, 5.58) | 0.0082 |
| No schooling | 23 (30) | 102 (8) | | | 11.7 (4.33, 31.61) | | <.0001 | | 3.07 (1.03, 9.16) | 0.0444 |  | 31 (24) | 81 (10) | 5.40 (2.52, 11.58) | <.0001 | 4.36 (1.91, 9.95) | 0.0005 |
| Number of years worked in agriculture (5-level variable) | | | | | | |  | |  |  |  |  |  |  |  |  |  |
| None | 36 (47) | | | 890 (72) | | *referent* |  | | *referent* |  |  | 20 (15) | 319 (39) | *referent* |  | *referent* |  |
| >0-4 | 5 (7) | | | 163 (13) | | 0.74 (0.29, 1.90) | 0.5247 | | 0.61 (0.23, 1.63) | 0.3203 |  | 15 (12) | 113 (14) | 2.12 (1.05, 4.29) | 0.0358 | 2.58 (1.22, 5.46) | 0.0133 |
| 5-9 | 9 (12) | | | 69 (6) | | 3.15 (1.46, 6.78) | 0.0035 | | 1.70 (0.73, 3.96) | 0.2213 |  | 9 (7) | 100 (12) | 1.44 (0.64, 3.26) | 0.3828 | 1.87 (0.79, 4.41) | 0.156 |
| 10-14 | 7 (9) | | | 50 (4) | | 3.38 (1.43, 7.95) | 0.0054 | | 1.78 (0.70, 4.51) | 0.2257 |  | 20 (15) | 101 (12) | 3.33 (1.73, 6.38) | 0.0003 | 3.64 (1.81, 7.32) | 0.0003 |
| 15+ | 19 (25) | | | 72 (6) | | 6.36 (3.48, 11.63) | <.0001 | | 1.73 (0.88, 3.39) | 0.1102 |  | 66 (51) | 186 (23) | 5.68 (3.33, 9.66) | <.0001 | 4.26 (2.40, 7.57) | <.0001 |
| Number of years worked in agriculture (3-level variable) | | | | | | |  | |  |  |  |  |  |  |  |  |  |
| None or <1 | 38 (4) | | | 943 (96) | | *referent* |  | | *referent* |  |  | 23 (6) | 347 (94) | *referent* |  | *referent* |  |
| 1-9 | 13 (7) | | | 182 (93) | | 1.77 (0.93, 3.39) | 0.0841 | | 1.27 (0.63, 2.55) | 0.5 |  | 21 (10) | 186 (90) | 1.70 (0.92, 3.16) | 0.0912 | 2.02 (1.05, 3.91) | 0.0366 |
| 10+ | 26 (18) | | | 122 (82) | | 5.29 (3.1, 9.02) | <.0001 | | 1.83 (1.01, 3.31) | 0.0464 |  | 87 (23) | 287 (77) | 4.57 (2.81, 7.43) | <.0001 | 3.71 (2.19, 6.29) | <.0001 |
| Number of times drank unregulated alcohol (lija) | | | | | | |  | |  |  |  |  |  |  |  |  |  |
| None | 76 (100) | | | 1243 (100) | | *referent* |  | | *referent* |  |  | 107 (82) | 732 (89) | *referent* |  | *referent* |  |
| ≥1 per week | 0 (0) | | | 1 (0) | | -- | -- | | -- | -- |  | 23 (18) | 87 (11) | 1.79 (1.09, 2.96) | 0.0225 | 1.96 (1.14, 3.36) | 0.0154 |
| Number of cigarettes smoked | | | | | |  |  | |  |  |  |  |  |  |  |  |  |
| None | 74 (97) | | | 1194 (96) | | *referent* |  | | *referent* |  |  | 99 (76) | 602 (74) | *referent* |  | *Referent* |  |
| ≥1 per day | 2 (3) | | | 50 (4) | | 0.64 (0.15, 2.67) | 0.5392 | | 0.77 (0.17, 3.42) | 0.7271 |  | 31 (24) | 217 (26) | 0.86 (0.56, 1.33) | 0.4983 | 0.88 (0.56, 1.40) | 0.6021 |
| Daily water consumption‡ | | | | | | | | |  |  |  |  |  |  |  |  |  |
| 0-4 | 19 (25) | | | 493 (40) | | *referent* | |  | *referent* |  |  | 15 (12) | 124 (15) | *referent* |  | *referent* |  |
| 5-8 | 26 (34) | | | 457 (37) | | 1.53 (0.84, 2.79) | | 0.1654 | 1.17 (0.62, 2.22) | 0.6348 |  | 32 (25) | 232 (28) | 1.14 (0.60, 2.19) | 0.6932 | 0.80 (0.39, 1.6) | 0.5206 |
| 9-12 | 21 (28) | | | 200 (16) | | 2.71 (1.43, 5.15) | | 0.0023 | 2.04 (1.01, 4.11) | 0.0472 |  | 37 (28) | 206 (25) | 1.48 (0.78, 2.82) | 0.2263 | 1.28 (0.64, 2.54) | 0.4834 |
| 13-52 | 10 (13) | | | 94 (8) | | 2.76 (1.24, 6.12) | | 0.0125 | 2.90 (1.22, 6.91) | 0.0161 |  | 46 (35) | 257 (31) | 1.47 (0.79, 2.74) | 0.221 | 1.36 (0.70, 2.65) | 0.362 |
| † Percents may not add up to 100 due to rounding.  *Logistic regression model adjusted for age (indicator-coded categorical variable), self-reported high blood pressure diagnosis, and self-reported diabetes diagnosis  § Calculated using the MDRD method[[11](#_ENREF_11)] eGFR variable truncated at 150  ‡Quartiles of ranked average number of glasses of water per day | | | | | | | | | | | | | | | | | |
